# The Gene Expression Deconvolution Interactive Tool (GEDIT): accurate cell type quantification from gene expression data

**DOI:** 10.1093/gigascience/giab002

**Published:** 2021-02-16

**Authors:** Brian B Nadel, David Lopez, Dennis J Montoya, Feiyang Ma, Hannah Waddel, Misha M Khan, Serghei Mangul, Matteo Pellegrini

**Affiliations:** Bioinformatics Interdepartmental Degree Program, Molecular Biology Institute, Department of Molecular Cellular and Developmental Biology, and Institute for Genomics and Proteomics, University of California Los Angeles, 610 Charles E Young Dr S, Los Angeles, CA 90095, USA; Bioinformatics Interdepartmental Degree Program, Molecular Biology Institute, Department of Molecular Cellular and Developmental Biology, and Institute for Genomics and Proteomics, University of California Los Angeles, 610 Charles E Young Dr S, Los Angeles, CA 90095, USA; Bioinformatics Interdepartmental Degree Program, Molecular Biology Institute, Department of Molecular Cellular and Developmental Biology, and Institute for Genomics and Proteomics, University of California Los Angeles, 610 Charles E Young Dr S, Los Angeles, CA 90095, USA; Bioinformatics Interdepartmental Degree Program, Molecular Biology Institute, Department of Molecular Cellular and Developmental Biology, and Institute for Genomics and Proteomics, University of California Los Angeles, 610 Charles E Young Dr S, Los Angeles, CA 90095, USA; Department of Mathematics, University of Utah, 155 1400 E, Salt Lake City, UT 84112, USA; Departments of Biology and Computer Science, Swarthmore College, 500 College Ave, Swarthmore, PA 19081, USA; Department of Clinical Pharmacy, USC School of Pharmacy, 1450 Alcazar Street Los Angeles, CA 90089, USA; Bioinformatics Interdepartmental Degree Program, Molecular Biology Institute, Department of Molecular Cellular and Developmental Biology, and Institute for Genomics and Proteomics, University of California Los Angeles, 610 Charles E Young Dr S, Los Angeles, CA 90095, USA; Department of Dermatology, David Geffen School of Medicine, University of California Los Angeles, 10833 Le Conte Ave, Los Angeles, CA 90095, USA

## Abstract

**Background:**

The cell type composition of heterogeneous tissue samples can be a critical variable in both clinical and laboratory settings. However, current experimental methods of cell type quantification (e.g., cell flow cytometry) are costly, time consuming and have potential to introduce bias. Computational approaches that use expression data to infer cell type abundance offer an alternative solution. While these methods have gained popularity, most fail to produce accurate predictions for the full range of platforms currently used by researchers or for the wide variety of tissue types often studied.

**Results:**

We present the Gene Expression Deconvolution Interactive Tool (GEDIT), a flexible tool that utilizes gene expression data to accurately predict cell type abundances. Using both simulated and experimental data, we extensively evaluate the performance of GEDIT and demonstrate that it returns robust results under a wide variety of conditions. These conditions include multiple platforms (microarray and RNA-seq), tissue types (blood and stromal), and species (human and mouse). Finally, we provide reference data from 8 sources spanning a broad range of stromal and hematopoietic types in both human and mouse. GEDIT also accepts user-submitted reference data, thus allowing the estimation of any cell type or subtype, provided that reference data are available.

**Conclusions:**

GEDIT is a powerful method for evaluating the cell type composition of tissue samples and provides excellent accuracy and versatility compared to similar tools. The reference database provided here also allows users to obtain estimates for a wide variety of tissue samples without having to provide their own data.

## Introduction

Cell type composition is an important variable in biological and medical research. In laboratory experiments, cell sample heterogeneity can act as a confounding variable. Observed changes in gene expression may result from changes in the abundance of underlying cell populations, rather than changes in expression of any particular cell type [1]. In clinical applications, the cell type composition of tissue biopsies can inform treatment. For example, in cancer, the number and type of infiltrating immune cells has been shown to correlate highly with prognosis ([2,3, 4]). Moreover, patients with a large number of infiltrating T cells are more likely to respond positively to immunotherapy [5].

For many years, cell flow cytometry via fluorescence-activated cell sorting (FACS) has been the standard method of cell type quantification. More recently, single-cell RNA sequencing (scRNA-seq) methods such as 10x Chromium, Drop-Seq, and Seq-Well have become available [6,7]. However, both approaches are hindered by significant limitations. FACS is cumbersome and expensive, and some sample types require hours of highly skilled labor to generate data. Similarly, scRNA-seq methods remain expensive for large sample studies. Additionally, cell types such as neurons, myocytes, and adipocytes are difficult for these technologies to capture owing to cell size and morphology.

Both FACS and single-cell methods have the potential to introduce bias because these technologies require that tissue samples be dissociated into single-cell suspensions. Many stromal cell types are tightly connected to one another in extracellular matrices. The procedures necessary to create single-cell suspensions can damage some cells, while others remain in larger clusters that are not captured or sequenced. Consequently, subtle differences in sample preparation can produce dramatically different results [8,9]. While FACS and single-cell methods can produce pure samples of each cell type, the observed cell counts may not accurately represent the cell type abundances in the original sample. Tools like SCDC and MuSiC use single-cell reference data to perform bulk deconvolution but require that multi-subject single-cell data be available for all the cell types of interest, which is not always the case [10,11].

During the past several years, digital means of cell type quantification, often referred to as cell type deconvolution or decomposition, have become a popular complement to FACS and single-cell approaches. However, these methods are still developing and are often hindered by limitations. For example, the tools MCP-Counter and xCell allow for deconvolution of a set of predefined cell types but do not support the inclusion of additional cell types or subtypes in a user-friendly manner [12,13]. CIBERSORT is slow to run on large datasets, particularly if signature genes are not specified, and provides reference data only for hematopoietic cell types [14].

To overcome some of the limitations of existing cell abundance estimation tools, we present the Gene Expression Deconvolution Interactive Tool (GEDIT). GEDIT uses gene expression data to accurately predict cell type composition of tissue samples. We have assembled a library of reference data from 11 distinct sources and use these data to generate thousands of synthetic mixtures. To produce optimal results, these synthetic mixtures are used to test and refine the approaches and parameters used by GEDIT. We compare the performance of GEDIT relative to other tools using 3 sets of mixtures containing known cell type proportions: 12 *in vitro* mixtures of immune cells sequenced on microarrays, 6 RNA-seq samples collected from ovarian cancer ascites, and 8 RNA-seq samples collected from blood. We also use GEDIT to deconvolute 2 sets of human tissue samples: 21 skin samples from patients with skin diseases and 17,382 samples of varied tissues from the Genotype-Tissue Expression (GTEx) database. Last, we apply GEDIT to the Mouse Body Atlas, a collection of samples collected from various mouse tissues and cell types. We find that GEDIT compares favorably to other cell type deconvolution tools and is effective across a broad range of datasets and conditions.

## Results

### Reference data

Reference data profiling the expression of purified cell types is a requirement for reference-based deconvolution. Methods that do not directly require reference data, such as non-negative matrix factorization, still require knowledge of expression profiles or marker genes to infer the identity of the predicted components. For the present study, we have assembled or downloaded a set of 11 reference matrices, each containing the expression profiles of 8–29 cell types (Table 1). These data sources span multiple platforms, including bulk RNA-seq, microarray, and scRNA-seq. Complete details on the sources and assembly of these matrices are described in the Methods [14–24].

**Table 1.** Library of reference data

Matrix	Species	Reference	Platform	No. of Cell types	Cell types
Human Skin Signatures	Human	[15]	Multi-Microarray	21	Immune
Human Body Atlas	Human	[16]	Affymetrix U133A/GNF1H	13	Immune
Human Primary Cell Atlas	Human	[17]	Affymetrix U133 Plus 2.0	26	Immune and stromal
BLUEPRINT^a^	Human	[18]	Bulk RNA-Seq	8	Immune
ENCODE^a^	Human	[19]	Bulk RNA-Seq	29	Mostly stromal
LM22	Human	[14]	Affymetrix Microarray	22	Immune
10x Single Cell Dataset^a^	Human	[20]	scRNA-seq	9	Immune
ImmunoStates	Human	[21]	Multi-Microarray	20	Immune
Tabula Muris	Mouse	[22]	scRNA-seq	12	Immune and stromal
Mouse Body Atlas	Mouse	[23]	Affymetrix Mouse Genome 430 2.0 Array	20	Immune and stromal
ImmGen	Mouse	[24]	Affymetrix Gene 1.0 ST	137	Immune with many subtypes

a Matrices assembled from source data as part of this project. All matrices are compatible with GEDIT and available on the GitHub repository [40].

### GEDIT algorithm

**Figure 1:**
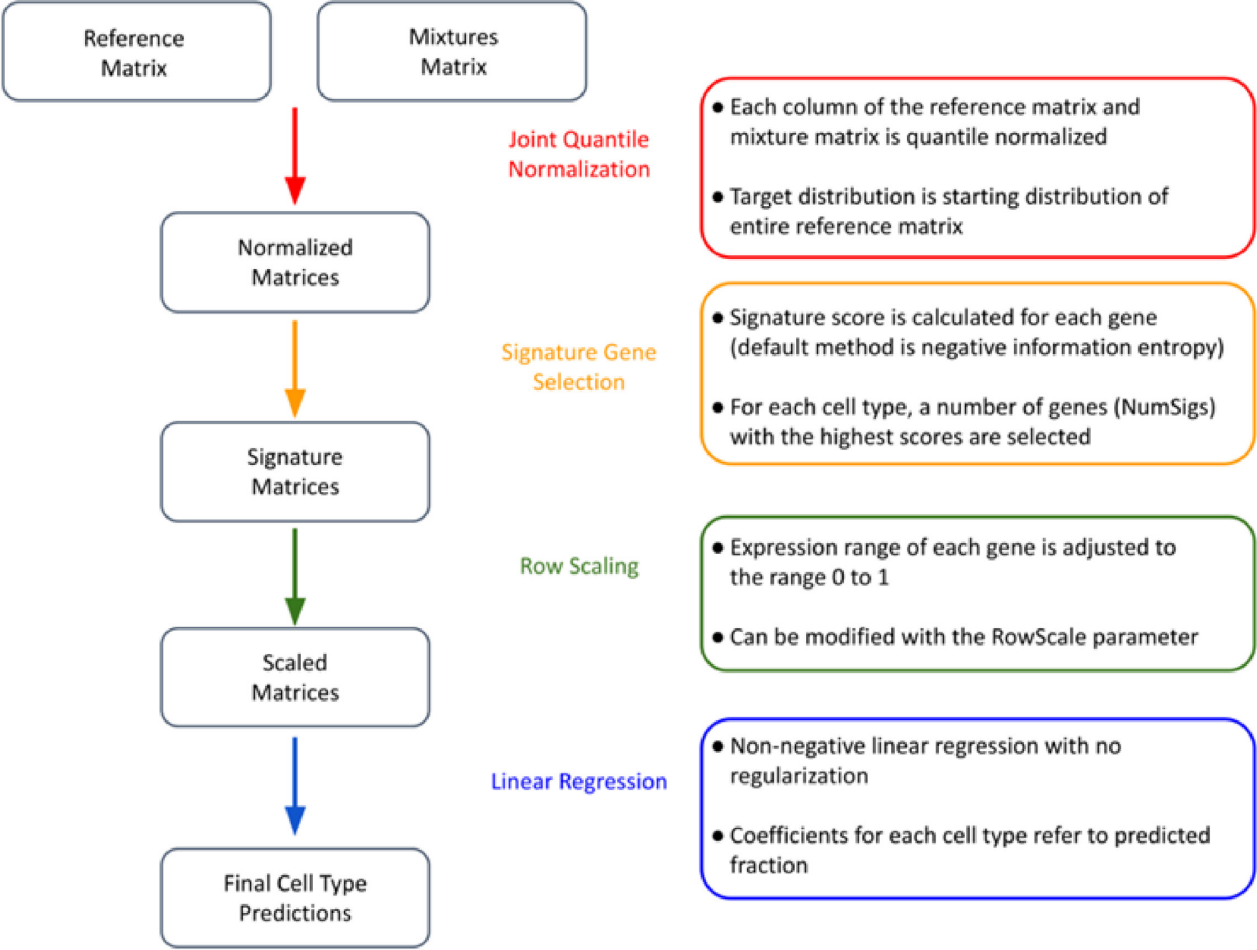
The GEDIT pipeline. The input matrices are quantile normalized then reduced to matrices containing only signature genes. Next, a row-scaling step serves to control for the dominating effect of highly expressed genes. Last, linear regression is performed, and predictions of cell type abundances are reported to the user.

GEDIT requires as input 2 matrices of expression values. The first contains expression data that are collected from the mixtures that will be deconvoluted; each column represents 1 mixture, and each row corresponds to a gene. The second matrix contains reference data, with each column representing a purified reference profile and each row corresponding to a gene. In a multi-step process, GEDIT uses the reference profiles to predict the cell type proportions of each submitted mixture (Fig. 1).

### Parameter tuning

**Figure 2:**
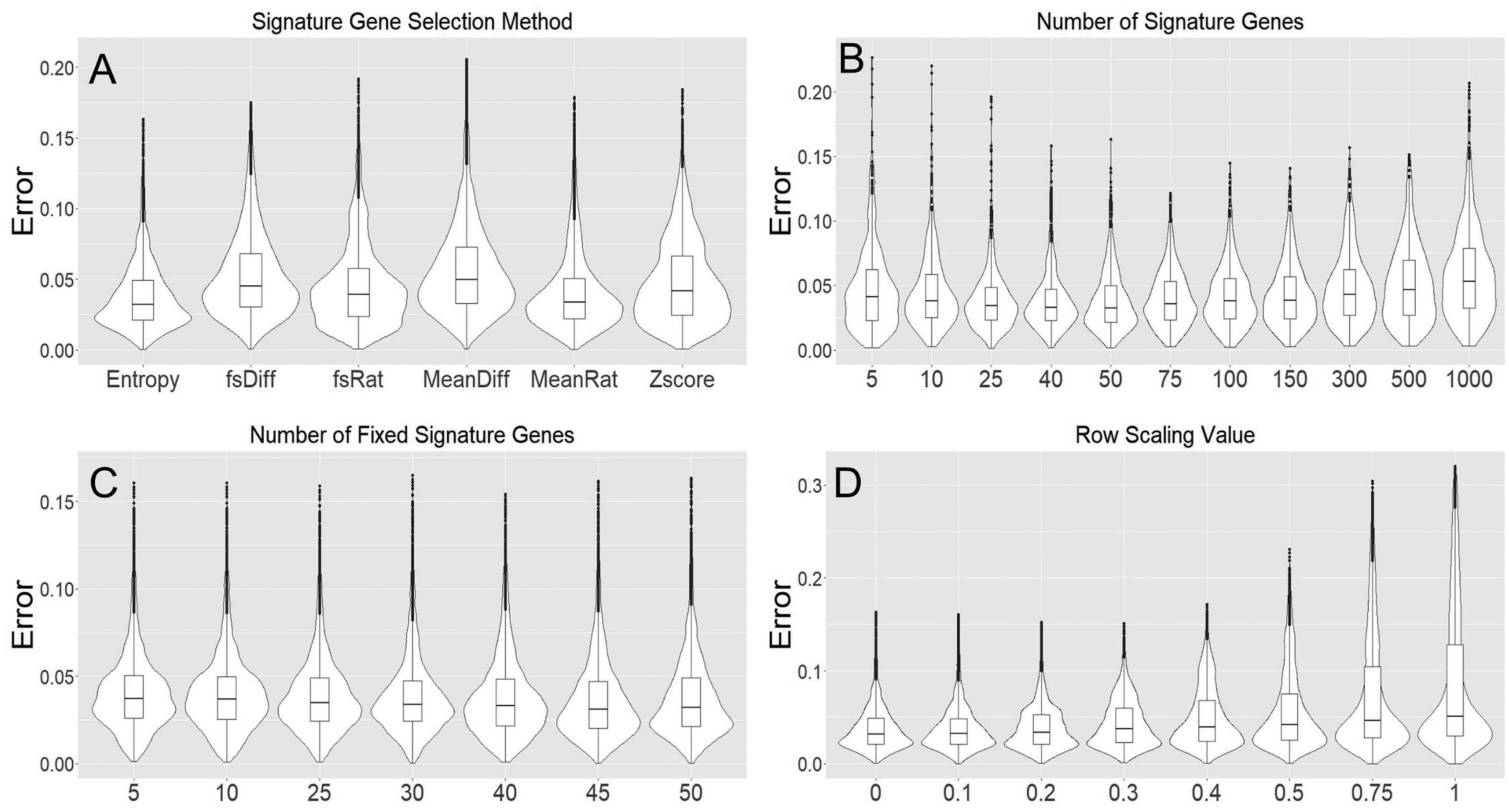
Effect of GEDIT parameter choices on accuracy of predictions in simulated experiments. A total of 10,000 simulated mixtures were generated, each using 1 of 4 reference matrices, with either 4, 5, 6, or 10 cell types being simulated. Boxplots display median error (central bar), the first and third quartiles (lower and upper boundaries of boxes), and outliers beyond 1.5 times the interquartile range (dots and whiskers). Violins display density of observations for each error value. Deconvolution was performed using a separate expression matrix than the one used to generate the mixtures. When not otherwise noted, we use the following parameters: signature selection method = entropy; number of signatures = 50; row scaling = 0; and number of fixed genes = number of signatures.

We generated a large number of synthetic mixtures *in silico* to test the efficacy of GEDIT and to assess how accuracy varies as a function of 4 parameter choices (SigMeth, NumSigs, MinSigs, RowScale, described in Table 2). We produced a total of 10,000 simulated mixtures of known proportions using data from 4 reference matrices: BLUEPRINT, the Human Primary Cell Atlas, 10x Single Cell, and Skin Signatures. We then ran GEDIT on these simulated mixtures and evaluated its performance while varying 4 parameter settings (Fig. 2) and other design choices. For this reason, these synthetic mixtures were not used to evaluate the performance of GEDIT relative to other tools. Instead, separate datasets were used for that purpose, as described in the section “Performance comparison to other deconvolution tools.” On the basis of these results, we selected default values for each parameter (SigMeth = Entropy, NumSigs = 50, MinSigs = 50, RowScale = 0). Full details on the generation of these simulations are described in the “Synthetic Mixture Generation" section of Supplementary Materials and Methods (See.

**Table 2:** GEDIT inputs include 2 matrices and 4 parameter settings

Input	Description	Allowed values	Default Value
RefMat	Matrix of purified cell types	N × M matrix; N is number of genes, M is number of cell types	NA
MixMat	Matrix of mixtures to be deconvoluted	N × P matrix; N is number of genes, P is number of mixtures	NA
SigMeth	Method of signature gene selection	Entropy, MeanRat, MeanDiff, ZScore, fsRat, fsDiff	Entropy
NumSigs	Mean number of signature genes per cell type	[1, 10,000]	50
MinSigs	Minimum number of signatures per cell type	[1, NumSigs]	=NumSigs
RowScale	Extent of per-row normalization	[0, 1.0]	0

RefMat is an expression matrix documenting the expression profiles of each cell type to be estimated. MixMat is an expression matrix documenting expression values for each sample to be deconvoluted. SigMeth determines the method by which signature genes are selected. NumSigs determines the total number of signature genes, whereas MinSigs sets the minimum number of signature genes for each cell type. RowScale refers to the extent to which expression vectors are transformed to lessen the dominating effect of highly expressed genes, with a value of 0 representing the most extreme transformation. Default values were determined by evaluating performance on a set of synthetic mixtures (Fig. 2). NA: not applicable.

### Preprocessing and quantile normalization

The first step in the GEDIT pipeline is to render the 2 matrices comparable. This is done by first excluding all genes that are not shared between the 2 matrices. Genes that have no detected expression in any reference cell type are also excluded because they contain no useful information for deconvolution. Both matrices are then quantile normalized, such that each column follows the same distribution as every other; this target distribution is the starting distribution of the entire reference matrix.

### Signature gene selection

GEDIT next identifies signature genes. Gene expression experiments can simultaneously measure tens of thousands of genes, but many of these genes are uninformative for deconvolution. Specifically, genes with similar expression levels across all cell types are of little use because observed expression values in the mixtures offer no insight into cell frequencies. Genes that are highly expressed in a subset of cell types are more informative, and we refer to these as signature genes. By using only signature genes rather than the entire expression matrix, the problem of deconvolution becomes more tractable and less computationally intensive. Moreover, identification of signature genes can be valuable to researchers for other applications (e.g., cell type assignment for scRNA-seq data).

To identify the best signature genes in a given reference matrix, GEDIT calculates a signature score for each gene. By default, this score is computed using the concept of information entropy. Information entropy quantifies the amount of information in a probability distribution, with highly uniform distributions having the highest entropy. The expression vector for each gene (i.e., the set of expression values across all cell types in the reference) is divided by its sum, such that the entries can be interpreted as probabilities. Information entropy is then calculated according to its mathematical definition (see Methods), and genes with the lowest entropy are selected as signature genes. Entropy is minimized when expression is detected only in a single cell type and maximized when expression values are equal across all cell types. Thus, by selecting genes with low entropy, we favor genes that are expressed in a cell type–specific manner. By default, 50 signature genes are selected for each cell type in the reference matrix. We chose 50 signature genes, and entropy as our scoring method, because it returned optimal results when run on 10,000 synthetic mixtures (see Fig. 2A and B).

We also evaluated the effect of accepting more signature genes for some cell types than others, depending on how many genes have low entropy. In this scheme, on average 50 signature genes are used per cell type. However, a fourth parameter is used, which specifies the minimum number of signature genes per cell type. After these have been selected, remaining signature genes are added based only on lowest entropy, regardless of cell type of maximal expression. We found that this parameter had minimal effect on accuracy when applied to synthetic mixtures (Fig. 2C). Therefore, this option is not used by default, although it can be specified by the user.

### Row scaling and linear regression

**Figure 3:**
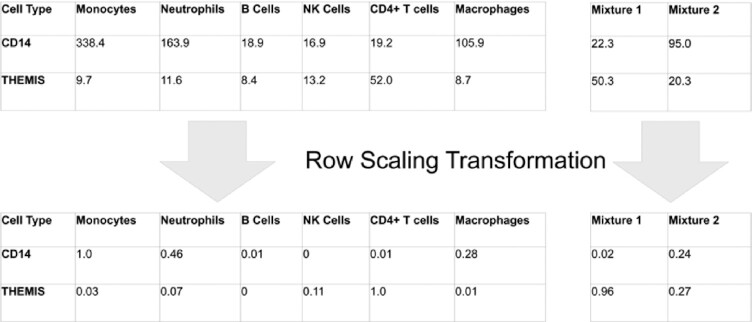
The “row scaling” transformation, as implemented by GEDIT. *CD14* and *THEMIS* are 2 examples of signature genes with drastically different magnitudes of expression. *CD14* is a signature gene for monocytes, and*THEMIS*, for CD4^+^ T cells. The original expression vectors are transformed, such that all values fall between 0 and 1.0, equalizing the effect of genes with varying magnitudes of expression.

One complication in the application of linear regression to gene expression data is the drastically different scale at which some genes are expressed. For example, *CD14* and *THEMIS* (Fig. 3) have both been identified as signature genes: *CD14* for monocytes and *THEMIS* for CD4-positive (CD4^+^) T cells. However, *CD14* is expressed at much higher levels in most cell types and will have a larger impact on the estimation of cell type composition, relative to *THEMIS*. In other words, the possible penalty resulting from a poor fit of *CD14* is much larger than the penalty from a poor fit of *THEMIS*.

To equalize the effect of each signature gene on the linear regression, we implement a transformation that we term “row scaling." Specifically, the range of all observed values for a particular gene (including reference cell types and samples) is adjusted such that the maximum value is 1.0 and the minimum value is 0. As a result, all genes have a comparable influence on the calculation of the linear regression solution, regardless of overall magnitude of expression. This transformation can be modulated by adjusting the row scaling parameter. By default, the value of this parameter is 0, and the transformation is applied as described above. Values between 0 and 1.0 are also allowed, which reduces the extent of the transformation (see Methods for details). Linear regression is then performed in R using the glmnet package, as described in the Methods.

### Performance comparison to other deconvolution tools

To assess the performance of GEDIT relative to other tools, we perform an experiment comparing GEDIT to 4 other deconvolution tools on datasets of known cell type content (CIBERSORT, DeconRNASeq, dtangle, and xCell [13,14,25,26]). Non-deconvolution tools like MCP-counter, SAVANT, and the DCQ algorithm are excluded from this study because they do not predict cell type fractions [12,27,28]. Tools that require single-cell data, such as MuSiC and CPM, are also excluded because this study is limited to tools that operate on bulk expression data [11,29]. See Table 3 for a summary of current bulk deconvolution methods.

**Table 3:** High-level characteristics of current cell type estimation tools

Tool	Publication	Custom reference	Approach	Output	No. of datasets	Cell types	Species
GEDIT	Refers to the present study	Yes	Deconvolution	Predicted fractions	11	Immune and stromal	Human, mouse
CIBERSORT	[14]	Yes	Deconvolution	Predicted fractions	1	Immune	Human
xCell	[13]	No	Marker genes	Predicted fractions	5	Immune and stromal	Human
dtangle	[26]	Yes, if marker genes specified	Deconvolution	Predicted fractions	0	NA	NA
DeconRNASeq	[25]	Yes	Deconvolution	Predicted fractions	0	NA	NA
DCQ	[28]	Yes	Deconvolution	Scores	3	Immune	Human, mouse
CIBERSORT (absolute mode)	[14]	Yes	Deconvolution	Scores	1	Immune	Human
SaVant	[27]	Yes, if marker genes specified	Marker genes	Scores	12	Immune and stromal	Human, mouse
MCP-Counter	[12]	No	Marker genes	Scores	NA	Immune and stromal	Human

Some tools accept custom references, which allows the tool to estimate the abundance of cell types not present in the default reference. Tools listed here take 1 of 2 approaches: they either perform deconvolution (most commonly regression) or calculate a score based on intensity of marker gene expression. Depending on the tool, the output can be interpreted as fractions corresponding to the abundance of each cell type, or as scores for each cell type that cannot necessarily be compared in an intercellular manner. NA: not applicable.

To perform this study, we use 3 datasets for which cell type fractions have been estimated using orthogonal methods. Two of these datasets were used in a recent benchmarking study [30]. Both are profiled using RNA-seq, and represent samples collected from either human cancer ascites or human blood [31,32]. In both cases, cell type fractions have been evaluated by FACS. The final dataset was prepared *in vitro* and consists of 6 cell types that were physically mixed together (in known proportions) to prepare 12 mixtures. These mixtures were then profiled using an Illumina HT12 BeadChip microarray. Adding to the previous benchmarking study, we also explore the effect of using 4 separate reference datasets: The Human Primary Cell Atlas, LM22, ImmunoStates, and a reference constructed from BLUEPRINT data. For each dataset, all tools (except xCell) were run 4 times, each time using a different reference matrix.

The optimal choice of reference matrix varies greatly depending on the exact combination of tool, dataset, and cell type. While using LM22 often produces the most accurate results, there are many exceptions. For instance, DeconRNASeq and GEDIT produce their best results for the blood dataset when using the BLUEPRINT reference. For the ascites data, several tools prefer ImmunoStates as the optimal reference choice. The best choice of reference is highly dependent on the nature of the input data and on the tool being used. In practice, researchers may wish to perform deconvolution multiple times—in each case using a separate reference matrix—and compare results for consistency.

**Figure 4:**
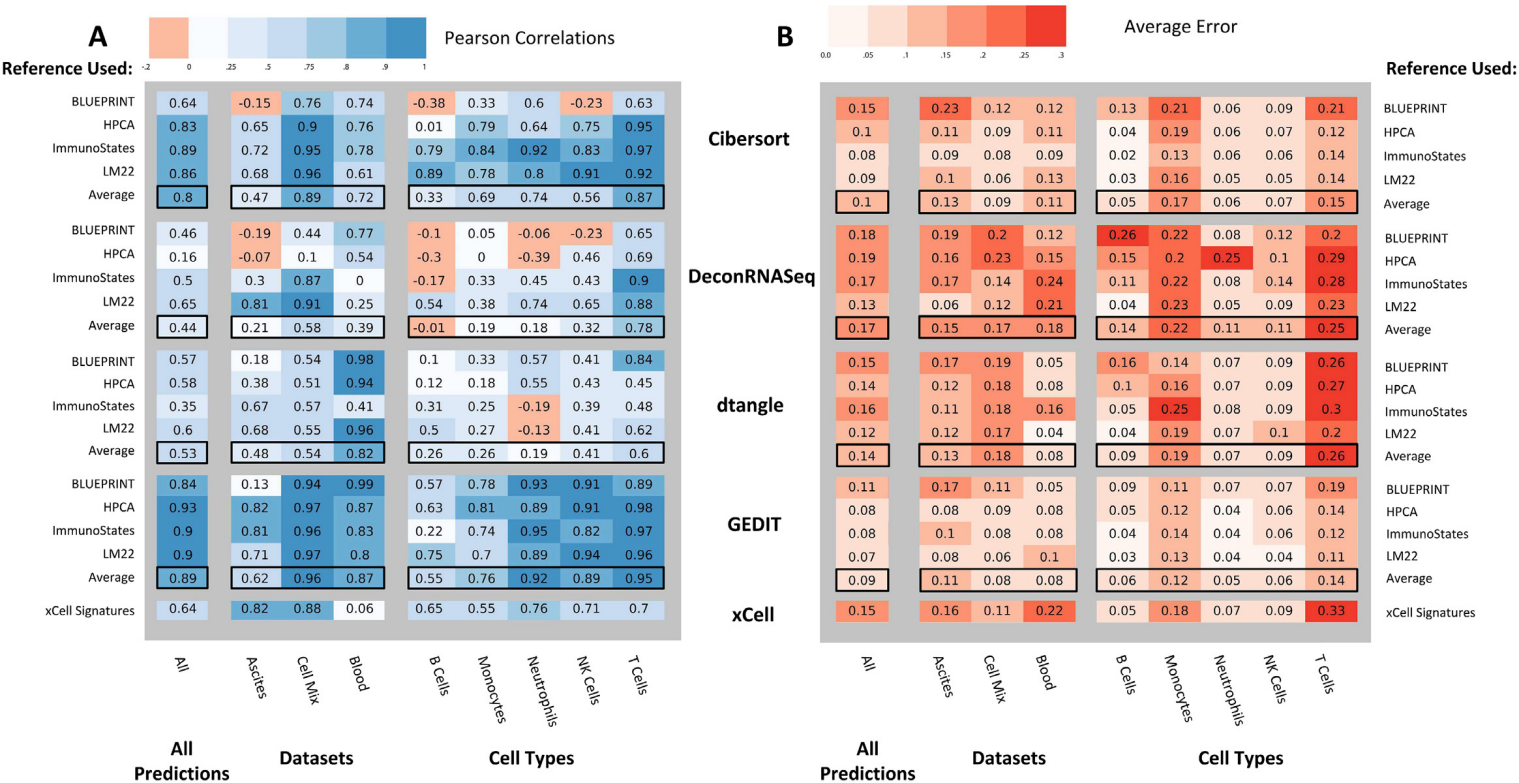
Performance of 5 deconvolution tools when applied to a set of 26 physical samples from 3 sources. Actual cell type fractions are either known due to controlled cell mixing (Cell Mix) or estimated by FACS (Ascites and Blood). In each instance, we calculate the correlation between actual cell type fractions and those predicted by deconvolution; deeper blues represent higher correlations (A). We similarly calculate mean error, with deeper reds representing higher error (B). We test 4 different reference datasets for each tool, and averaged correlations across these 5 cases are shown in boxes. We calculate correlations for each cell type (right 5 columns), for each of the 3 mixtures (middle 3 columns), and for all predictions regardless of cell type or data source.

Compared to the other tools, GEDIT produced robust and consistently accurate results (Fig. 4, Supplementary Figs S1 and S2). For many tools, the quality of predictions varies greatly depending on the cell type, dataset, or choice of reference matrix. When results are averaged across the 4 possible reference choices, GEDIT produces the minimum error and maximum correlation for all 3 datasets. This result suggests that GEDIT is a strong choice when researchers are using novel references matrices that have not been curated or tested.

Last, we perform an evaluation of runtime required for each tool. We randomly select batches of 100, 200, 500, 1,000, and 2,000 samples from the GTEx database, and measure CPU time required to deconvolute these batches for each tool. The runtimes of GEDIT, dtangle, and DeconRNASeq scale well with increasing input size, taking ≤20 minutes (Supplementary Fig. S3). For larger input sizes, CIBERSORT can take >1 hour.

### Comparison to single-cell methods

We also compare GEDIT to 2 contemporary deconvolution tools that use single-cell data as their reference, namely, SCDC and MuSiC [10,11]. We reproduce the steps provided by the SCDC authors to generate 2 sets of 100 simulated pancreatic mixtures. These data are created *in silico* using single-cell data from 2 recent studies, and contain randomized mixtures of α-, β-, γ-, and δ-cells from pancreatic islets [33,34]. Data from a third study were used as a reference for all 3 tools, and similarly contain α-, β-, γ-, and δ-cells [35]. In the case of SCDC and MuSiC, these data are used in their original single-cell form. For GEDIT, pseudo-bulk expression profiles for each of the 4 cell types were created by averaging the expression values of each member cell (e.g., expression of all α-cells were averaged to create an α-cell reference profile).

**Figure 5:**
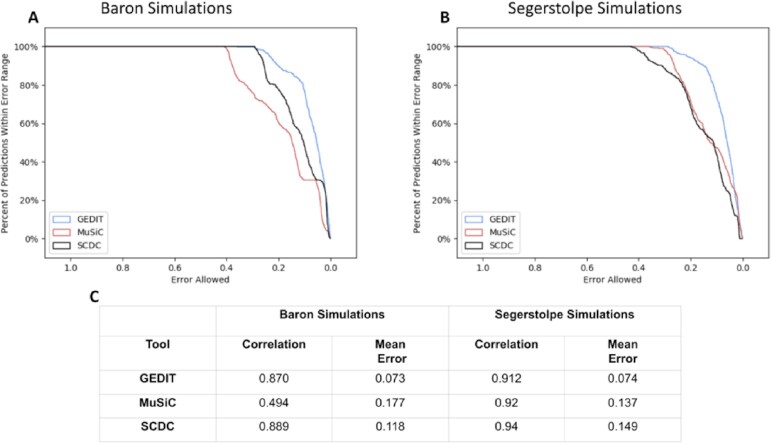
Performance of GEDIT and 2 single-cell deconvolution tools when applied to simulated pancreatic islet mixtures. Two sets of 100 synthetic mixtures were used, each set from a separate data source of scRNA-seq α-, β-, γ-, and δ-cells [33,34]. Protocols developed by the SCDC authors were used to create synthetic mixtures *in silico*. Data from a third source were used as a reference [35]. (A, B) Error distribution plots for each of the 2 datasets. Each point on a graph represents the percentage of predictions (y-axis) that are accurate within a particular error range (x-axis). (C) Summary statistics of predictions for each tool and dataset. Pearson correlation and mean average error between predicted and actual cell type fractions are shown.

The results of GEDIT compare favorably to the 2 single-cell tools (Fig. 5). GEDIT produces the lowest error on the 2 sets of simulated mixtures by a substantial margin. Based on the metric of correlation between predicted and actual fractions, GEDIT produces results comparable to SCDC, and either comparable or superior to MuSiC, depending on the set of mixtures (Fig. 5c, Supplementary Fig. S4). Thus, by using the methodology of averaging cell clusters in the reference dataset, GEDIT can be applied to datasets suitable for SCDC or MuSiC. We also apply 3 other bulk deconvolution tools to this same dataset and show that GEDIT provides the best performance of the 4 (Supplementary Fig. S5).

### Skin expression data

**Figure 6:**
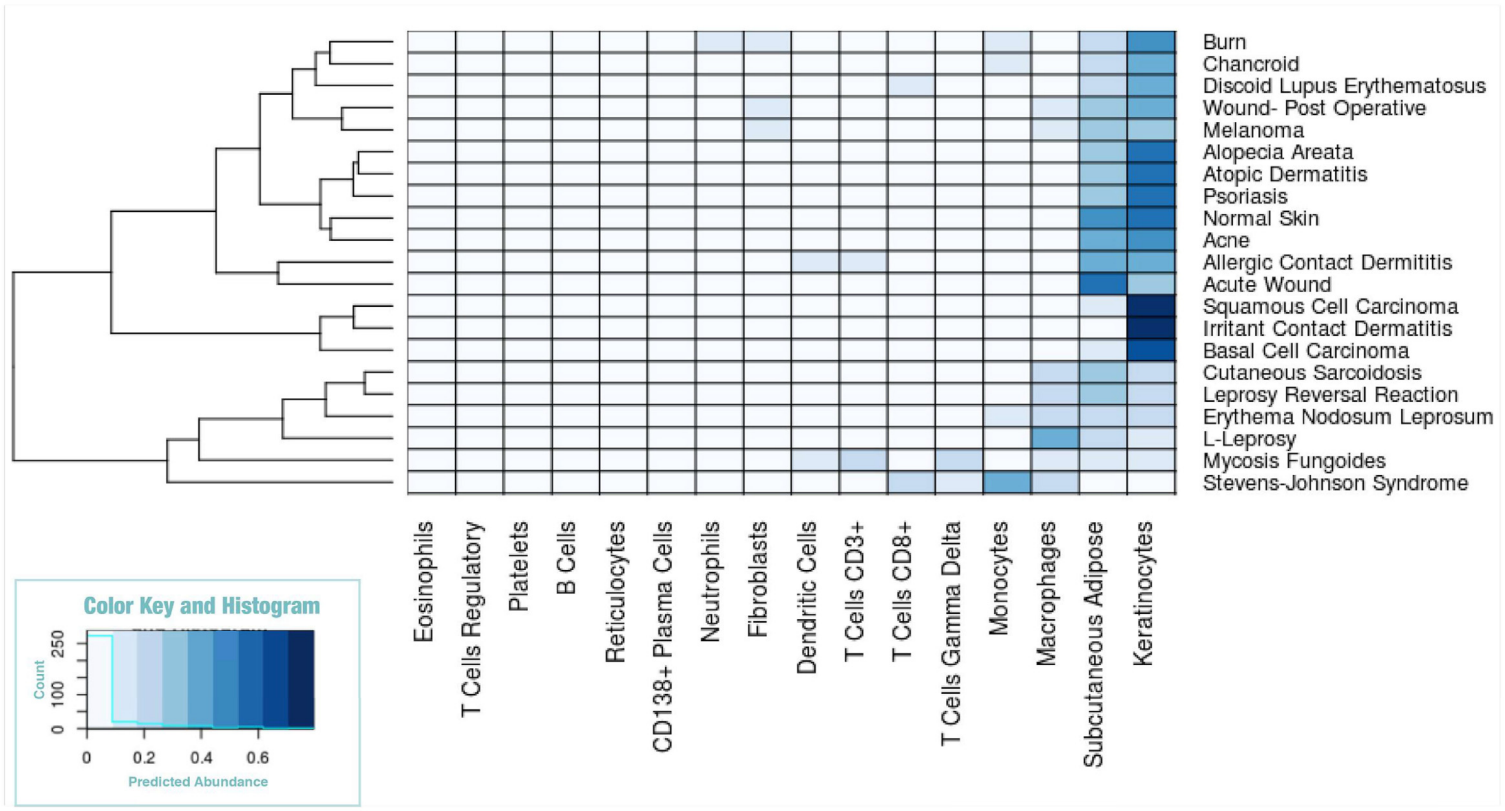
GEDIT predictions for 21 samples of various skin diseases. GEDIT correctly identifies keratinocytes and subcutaneous adipose as the most common cell. Deviations from this pattern correspond to disease biology. Blister fluid from Stevens-Johnson syndrome is predominantly immune cells. L-leprosy and leprosy reversal reaction result in large numbers of macrophages. Mycosis fungoides is a T-cell lymphoma.

We further validate GEDIT by using it to deconvolve a set of skin biopsies from humans with a variety of skin diseases [13]. The exact cell type composition of these samples is unknown, but we have reasonable expectations based on skin and disease biology. For example, macrophages are known to be abundant in granulomas of leprosy lesions, and Stevens-Johnson syndrome produces blisters that fill with large numbers of monocytes [36,37]. We find that, in all cases, predictions made by GEDIT conform well with these biological expectations. Keratinocytes are highly predicted in most cases, as one would expect with skin samples (Fig. 6). Deviations from this pattern correspond with disease biology. Monocytes are highly predicted in Stevens-Johnson syndrome, as are macrophages in the 3 leprosy samples, and T cells in the mycosis fungoides (T-cell lymphoma) sample. Three other deconvolution tools were also applied to this dataset, and predictions follow similar patterns (Supplementary Fig. S6).

### Application of GEDIT to mouse data

**Figure 7:**
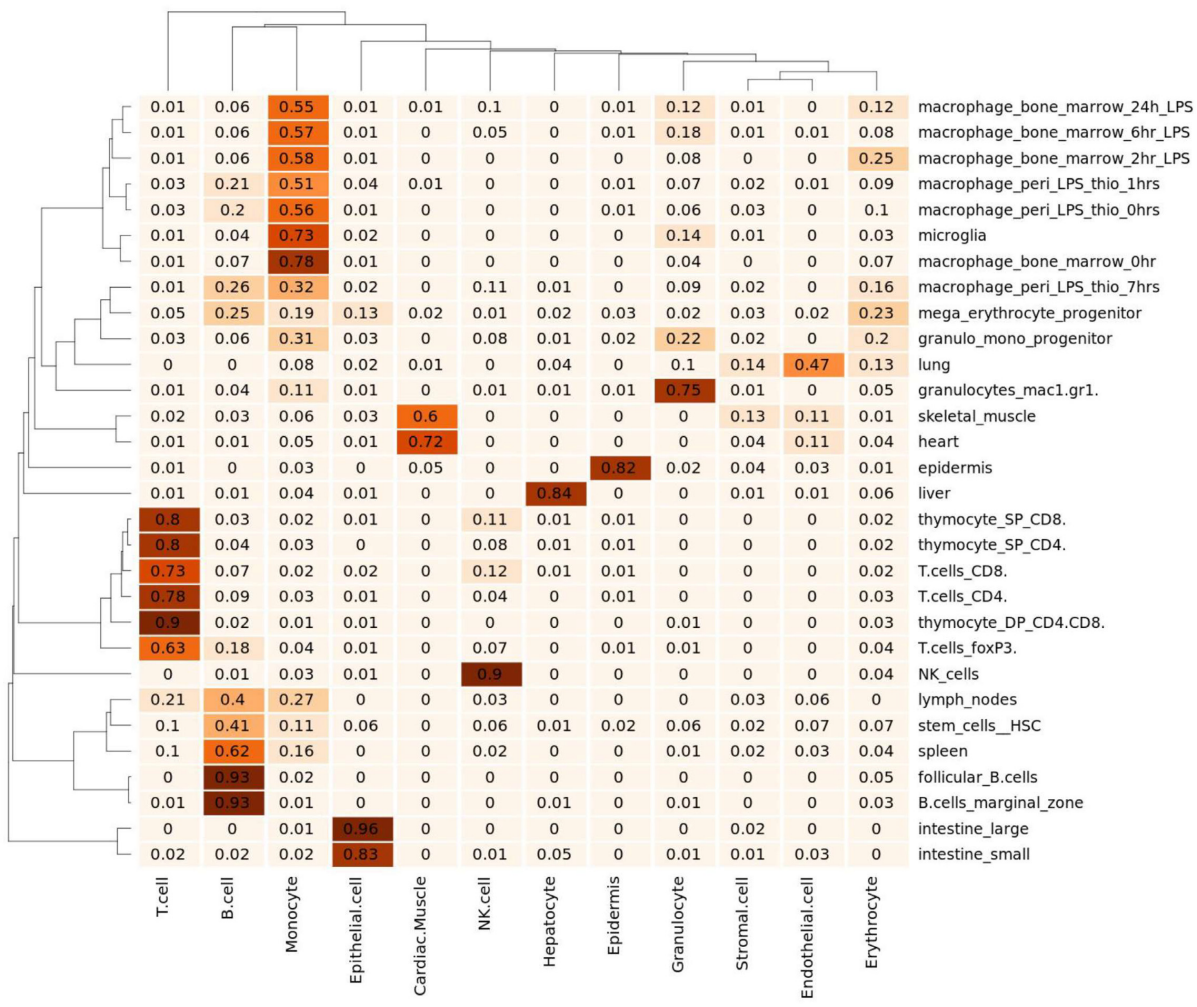
GEDIT predictions on 30 samples collected from various mouse tissues and cell types (Mouse Body Atlas [23]). Predictions largely conform with tissue and cell biology.

GEDIT can be used to decompose data from any organism for which reference data are available. Here, we demonstrate the efficacy of GEDIT when applied to the Mouse Body Atlas, a collection of tissue and cell type samples collected from mice (GEO: GSE10246) [23]. As reference data, we assembled a matrix of 12 cell types using single-cell data from the Tabula Muris [22]. GEDIT correctly infers the identity of purified cell types, including 6 samples that consist of either pure NK cells, B cells, T cells, or granulocytes (Fig. 7). An entry for macrophages is not available in the reference used, but most macrophage samples are identified as monocytes, which is the most similar cell type present in the reference matrix. For more complex tissues, GEDIT predicts cell type fractions that correspond to the biology of the samples. Hepatocytes are predicted to be highly prevalent in the liver sample (84%) and are not predicted in any other sample (<5% in all cases). Similar patterns hold for keratinocytes in the epidermis, epithelial cells in 2 intestinal samples, and cardiac muscle cells in heart and muscle samples. Three other deconvolution tools were also applied to this dataset, and predictions follow similar patterns (Supplementary Fig. S7).

### Deconvolution of GTEx Database

**Figure 8:**
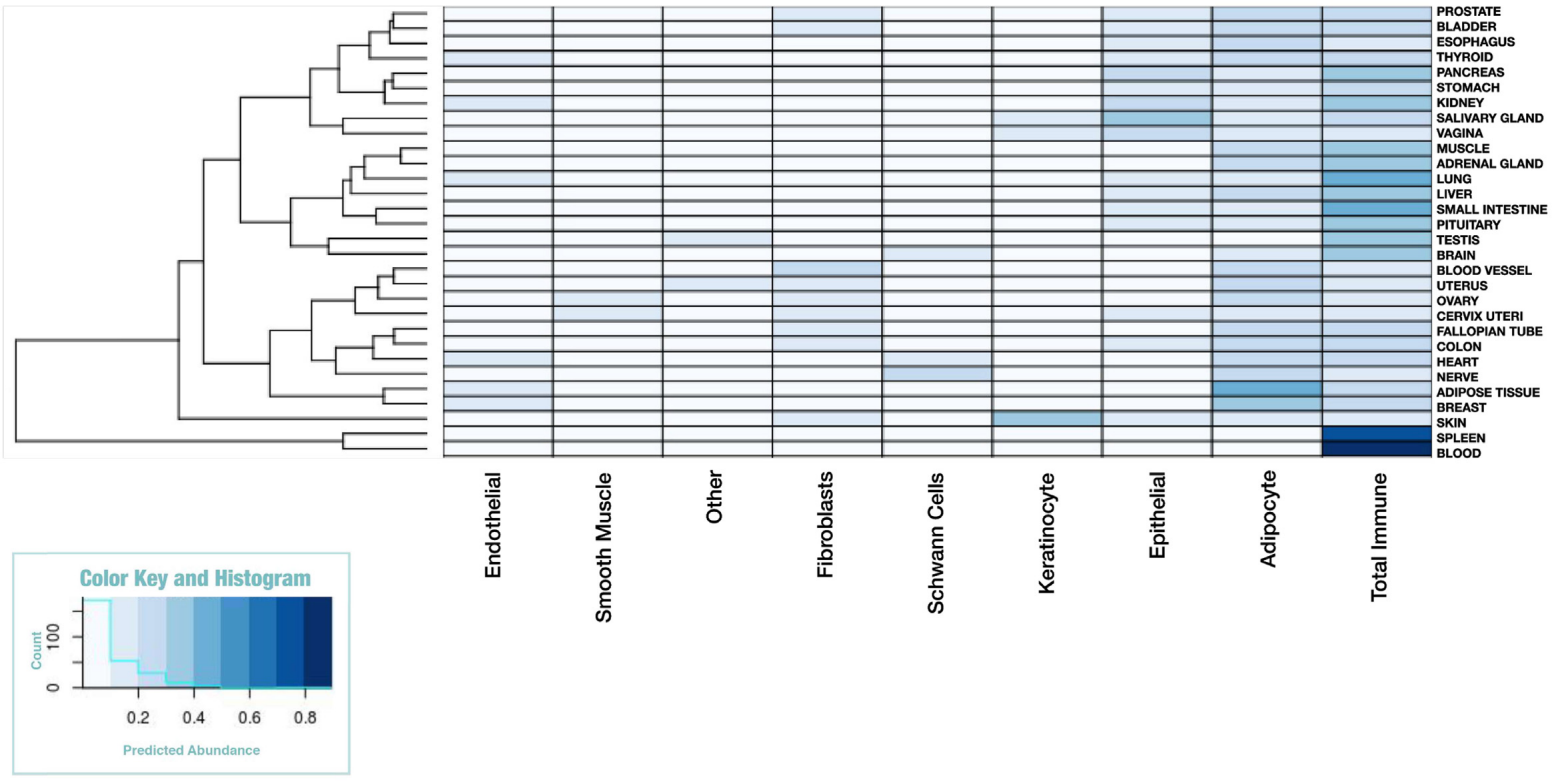
GEDIT cell type predictions when applied to 17,382 samples from the GTEx database. Here, predictions have been averaged for each tissue of origin (see Supplementary Methods for additional details).

To assess the use of GEDIT across very large datasets, we applied the tool to 17,382 GTEx RNA-seq samples collected from various tissues and accessed via the GTEx portal [38]. However, no single reference contained all cell types expected to be present and combining references from separate experiments and platforms is problematic (Supplementary Figs S8–S10). Therefore, we took an alternate approach by performing deconvolution 3 times using 3 separate references (BlueCode, Human Primary Cell Atlas, Skin Signatures). We then combine these outputs by taking their median value; after normalization, we treat this median value as a final cell type estimate (see “Deconvolution of GTEx Database" section in Supplementary Materials and Methods for more details). While this approach did enable predictions spanning a larger number of cell types than are present in any 1 reference matrix, it must be noted that it is not a proper substitute for a single unified reference (Fig. 8).

These predictions largely conform to biological expectations. For example, immune cells are predicted to have high abundance in blood and spleen, adipocytes in adipose tissue, Shwann cells in nerve and heart, and keratinocytes in skin. Each of these patterns matches expectations of which cell types should be present in these tissues. However, neither cardiac myocytes nor smooth muscle are highly abundant in GTEx muscle samples. This is likely because the GTEx samples are collected from skeletal muscle, which is known to have an expression profile that is distinct from that of cardiac and smooth muscle.

### GEDIT availability

GEDIT can be run online and source code, associated data, and relevant files are available via GitHub [40]. We provide access to the tool, a set of varied reference data, and 2 sample mixture matrices. The website automatically produces a heat map of predicted proportions for the user, as well as a .tsv file. The user also has access to the parameter choices of GEDIT (signature gene selection method, number of signature genes, row scaling).

## Methods

### GEDIT algorithm

#### Signature gene selection

During signature gene selection, we automatically exclude genes with zero detected expression in half or more of cell types. Observed expression values of exactly zero are often the result of either technical artifacts or resolution issues. Using such genes as signatures can result in inaccurate and highly unstable results, particularly when working with scRNA-seq derived data. As an additional safeguard, we treat all remaining expression values of zero as the lowest observed non-zero value in the matrix. Implementing this change has minimal effect on most genes but prevents genes with resolution issues from achieving artificially high scores. We consider this transformation valid because values of zero generally do not represent zero expression but rather an expression level below the detection limit of the technology used.

For any given gene, a scoring method takes as input the vector of the expression values across all reference cell types, and outputs a score. A gene is considered a potential signature gene in cell type *X* if it is expressed more highly in *X* than any other cell type. For each cell type, we keep only the *N* genes with the highest scores, where *N* is the NumSigs parameter.

Information entropy (*H*) is calculated using the following formula:

(1) \begin{equation*} H\,\, = \,\, - \mathop \sum \nolimits_1^i \left[ {{p_i}*\left( {{p_i}} \right)} \right], \end{equation*}

where *p_i_* is the probability of the *i*th observation. To apply this to expression values, we convert the vector of expression values into a vector of probabilities by dividing by its sum. In an equal mixture of each cell type, the *i*th probability can be interpreted as the fraction of transcripts originating from the *i*th cell type.

#### Row scaling

During this step, we apply a transformation on the expression values for each gene. Each gene has measured expression in *N* purified cell types and *M* samples. Each of these values, *X*_old_, is transformed according to the following formula:

(2) \begin{equation*} {X_{\mathrm{new}}} = \,\,({X_{\mathrm{old}\,\,}} - \mathrm{Min})/\left( {\mathrm{Max} - \mathrm{Min}} \right)\,\,*\,\,\mathrm{Max}^p, \end{equation*}

where Min is the minimum of all *M* + *N* original values, Max is the maximum of those values, and *p* is a tunable parameter with natural range *p ∈* [0, 1.0]. This procedure produces values between the range of 0 and Max^*p*^.

#### Linear regression

Non-negative linear regression was performed using the glmnet package in R. The glmnet function is used with lower.limits = 0, α = 0, λ = 0, intercept = FALSE. These settings perform a linear regression where all coefficients are non-negative, and with no regularization and no intercept term.

## Reference Data

### BLUEPRINT reference dataset

A total of 35 gene counts files were downloaded from the BLUEPRINT database, all collected from venous blood [18]. This included entries for CD14^+^, CD16-negative (CD16^−^) classical monocytes (5 samples), CD38^−^ naive B cells (1), CD4^+^, α-β T cells (8), central memory CD4^+^, α-β T cells (2), cytotoxic CD56-dim NK cells (2), macrophages (4), mature neutrophils (10), and memory B cells (1). When 2 or more transcripts appeared for a single gene, the transcript with the highest average expression was selected and others were excluded. Genes with no detected expression in any sample were also excluded, and then each sample was quantile normalized. Samples generally clustered by cell type, but we excluded 1 CD4^+^ α-β T cell. Replicates for each cell type were then collapsed into a single entry by taking the median value for each gene.

### ENCODE reference dataset

A total of 106 transcript quantification files were downloaded from the Encyclopedia of DNA Elements (ENCODE) database [19]. These included all RNA-seq experiments collected from adult primary cells, excluding 4 with warnings. Warnings indicated that 3 samples were hindered by low replicate concordance and 1 sample by low read depth, and these samples were excluded. All samples were processed by the Gingeras Lab at Cold Spring Harbor and mapped to GRCH38.

The samples were quantile normalized and clustered. In cases where multiple transcripts were measured for a single gene, the expression of that gene was calculated as the sum of all transcripts. At this time, 18 additional samples were excluded because they did not cluster with their replicates. On the basis of sample descriptions and data clustering, we found that the remaining 88 samples represented 28 unique cell types. We produced an expression profile for each cell type by merging all samples of that cell type via median average. For example, a cluster of 19 samples were labeled as endothelial cells (collected from various body locations) and were merged into a single entry termed canonical endothelial cells. This dataset spans a wide range of stromal cell types (e.g., smooth muscle, fibroblast, epithelial) but contains only a single entry for blood cells, which are labeled mononuclear cells.

We also combined the ENCODE and BLUEPRINT reference matrices into a single reference matrix, which we call BlueCode. We combined, then quantile normalized, the columns of both matrices. Possible batch effects in this combined matrix have not been fully evaluated.

### 10x Genomics Reference dataset

We obtained single-cell expression data for 9 varieties of immune cells from the 10x Genomics website [20]. This included ≥2,446 cells for each cell type, and ≥7,566 cells for all cells other than CD14^+^monocytes. For each cell type, expression values for all cells were mean averaged to form an expression profile.

### Tabula Muris reference dataset

We downloaded from the Tabula Muris single-cell data for 12 clusters of mouse cell types. For each cluster, we averaged all cells of that cluster to produce a reference profile for the corresponding cell type.

### Other reference datasets

Other datasets used in this project were obtained from their corresponding publications or GEO repositories. This includes a reference matrix of human skin signatures, the Human Body Atlas, the Human Primary Cell Atlas, LM22, ImmunoStates, the Mouse Body Atlas, and ImmGen [14–17,21,23,24].

### Skin disease data

We obtained expression data from 21 skin biopsies, collected from human patients with a variety of skin diseases. These data originally came from a wide range of sources and platforms and were compiled into a single dataset by previous work [39].

### GTEx data

GTEx data for 17,382 samples were obtained from the GTEx portal [38]. We ran GEDIT on all samples 3 times, each time using a different reference matrix (BlueCode, the Human Primary Cell Atlas, and Skin Signatures). For each cell type, we calculated our initial estimate as the median estimate across the 3 sets of predictions (or fewer, if that cell type is missing 1 of 2 of the reference matrices). Last, for each sample we divided the vector of predictions by its sum, such that the final predictions sum to 100%.

## Multi-Tool Performance Evaluation

### *In vitro* immune cell mixture

Combinations of 6 immune cells (neutrophils, monocytes, NK cells, B cells, and CD4^+^ and CD8^+^ T cells) were mixed together and sequenced using an Affymetrix array. Whole-blood samples from healthy human donors were supplied with informed consent through a sample-sharing agreement with the UCLA/CFAR Virology Core Lab. CD4^+^ T cells, CD8^+^ T cells, B cells, and NK cells were isolated using Stem Cell Technologies (Vancouver, BC, Canada) RosetteSep negative selection. Neutrophils were positively selected through the EasySep approach, according to the manufacturer's specifications. Cells were then counted by hemocytometer and added at defined percentages to a total cell count of 2 million cells to create 6 different mixtures. Subsequently cells were processed for RNA isolation by AllPrep DNA/RNA. Illumina HT12 BeadChip microarray was performed by the UCLA Neuroscience Genomics Core. Data were normalized by quantile normalization through the R “normalize.quantiles” function.

### RNA-seq mixtures used for tool evaluation

We also obtained 2 datasets used in a recent benchmarking study [30]. The first dataset is composed of 3 RNA-seq samples, each with 2 technical replicates that represent biopsies of ovarian cancer ascites [32]. The second dataset is composed of RNA-seq collected from the blood of healthy individuals, some of whom had recently received an influenza vaccine [31]. These data were downloaded from the GitHub site for the benchmarking paper, which also contained FACS estimates for 6 cell types for the ascites data (B cells, dendritic cells, NK cells, T cells, macrophages, neutrophils) and 5 cell types for the blood data (B cells, dendritic cells, T cells, monocytes, NK cells) [30]. However, because dendritic cells were never present at >3.5% abundance, we did not evaluate performance for this cell type.

### Tools

We installed and ran GEDIT, CIBERSORT, DeconRNASeq, and dtangle on the hoffman2 computational cluster at UCLA. xCell was run using the online interface [13]. The default choice for gene signatures (xCell = 64) was used. The RNA-seq option was selected for the 2 RNA-seq datasets (blood and ascites) but not for the *in vitro* dataset, which was sequenced on microarray.

xCell produces 67 output scores, 7 of which were used in this study. These were the entries labeled “B-Cells,” “Macrophages,” “Monocytes,” “NK cells,” “Neutrophils,” “CD4+ T cells,” and “CD8+ T Cells.” As suggested by the xCell authors, the outputs for CD4^+^ and CD8^+^ T-cell subtypes were summed to produce a final output for total T cells.

### Reference data

We evaluated the performance of the 4 reference-based tools (GEDIT, CIBERSORT, DeconRNASeq, and dtangle) using each of 4 choices of reference matrix (LM22, ImmunoStates, BLUEPRINT, and the Human Primary Cell Atlas). The BLUEPRINT and Human Primary Cell Atlas reference matrices differ from ImmunoStates and LM22 in that they contain tens of thousands of genes, many of which should not be considered signature genes. This contrasts to ImmunoStates and LM22; each reference matrix contains <600 genes, which have been specifically identified as signature genes by previous work [14,21]. We include both forms of reference matrices in order to evaluate the input requirements of the tools studied.

Depending on the choice of reference matrix, reference-based tools often produce multiple outputs for some cell types, each representing a cell subtype. This includes B cells (naive and memory), monocytes (CD14 and CD16), NK cells (resting and active), and T cells (many subtypes including varieties of CD4 and CD8). In each case, the outputs for each subtype were summed to produce a total score for each greater cell type.

## Discussion

GEDIT is an expression-based cell type quantification tool that offers unique flexibility and accuracy in a wide variety of contexts. Using both simulated and experimental data, we demonstrate that GEDIT produces high-quality predictions for multiple platforms, species, and a diverse range of cell types, outperforming other tools in many cases. We include in the software package a comprehensive library of reference data, which facilitates application of GEDIT to a wide range of tissue types in both human and mouse. GEDIT can also accept reference data supplied by the user, which can be derived from bulk RNA-seq, scRNA-seq, or microarray experiments. GEDIT represents a competitive addition to the suite of existing tissue decomposition tools while maintaining flexibility and performance robustness.

As part of this project, we performed a study in which we compared the performance of several deconvolution tools using multiple metrics. Unlike previous evaluation studies, we explored the effect of reference choice by running tools multiple times with reference data from different sources. Choice of optimal reference has a large impact on the accuracy of many tools, but GEDIT provided robust performance and accurate estimates for many possible reference choices. While all efforts were taken to perform this comparison in an unbiased manner, we note that development of the tool was still underway when the first comparisons were made. All code and inputs used to reproduce this study are included in the GitHub repository [40], with the exception of CIBERSORT code, which is limited by copyright.

The high performance of GEDIT is due to 2 key innovations. First, signature gene selection by information entropy serves to select genes that are the most informative for deconvolution. Second, the row scaling step aims to equally weight all signature genes into the final estimate, even those with comparatively low expression. In addition, the flexibility of GEDIT and the diverse set of reference matrices that we provide allows GEDIT to be easily applied in a wide range of circumstances.

The output of GEDIT represents the fraction of mRNA originating from each cell type. This is an effective measure of the transcriptional contribution of each cell type in a mixture. However, in cases where some cell types consistently produce more or less mRNA per cell, this measure may not represent cell counts. Data capturing the average mRNA content per cell is becoming more widely available in the form of single-cell experiments and could in principle be used to convert our fractions into cell counts.

When extensively applied to several large public datasets, GEDIT produces predicted cell type fractions that conform with biological expectations. When used to decompose skin biopsies, GEDIT finds keratinocytes to be the most abundant cell type. Variations in the abundance of other cell types conform to expected immune responses across diseases. Similarly, cell type predictions of GTEx samples are concordant with our expectations of the dominant cell types across tissues. Schwann cells, keratinocytes, adipose cells, and immune cells are found to be most abundant in nerve, skin, adipose tissue, and blood, respectively.

Single-cell RNA-seq is an emerging approach to study the composition of cell types within a sample. Owing to biases associated with the capture of different cell types, these methods are not always capable of accurately quantifying cell type populations [8]. However, the pure reference profiles produced by existing methods can be used by GEDIT to generate accurate estimates of cell type populations. Thus, GEDIT circumvents some of the biases associated with the preparation of samples for both scRNA-seq and FACS. GEDIT is freely available and therefore an economical option for researchers, particularly those who profile expression data for other purposes.

GEDIT produces accurate results when tested on mixtures of human immune cells. Compared to other tools, GEDIT produces the lowest error in the majority of scenarios in the studied mixtures. GEDIT provides increased flexibility over previously developed tools, as we provide a set of reference matrices for varied cell types for both mouse and human datasets.

GEDIT provides unique advantages to researchers, especially in terms of cell type, species, and platform flexibility, and constitutes a useful addition to the existing set of tools for tissue decomposition. Our efficient decomposition methodology has been extensively optimized, and we find that it performs robustly across a broad range of tissues in both mouse and human datasets. Our future work will extend reference matrices to facilitate application of GEDIT on varied bulk gene expression datasets.

## Availability of Source Code and Requirements

- Project name: GEDIT
- Project home page: https://github.com/BNadel/GEDIT
- Programming languages: Python 2.0, R
- Other requirements: numpy, glmnet
- Operating systems: Linux
- License: MIT

## Data Availability

All data used in this article are freely available on GitHub [40], as well as their original sources. Code for DeconRNASeq was obtained as an R package from the CRAN repository. Code for CIBERSORT was obtained by requesting it via the web portal [14], and code for dtangle from the project's GitHub page [26].

Reference data are also available from their original sources. Most datasets can be found on project website pages or from public databases. These include BLUEPRINT [18], ENCODE [19], the Human Primary Cell Atlas [17], LM22 [14], 10x Genomics [20], Tabula Muris [22], the Mouse Body Atlas [23], and ImmGen [24]. Some reference matrices were obtained as supplementary files from the publications listed in Table 1.

Expression values for the blood and ascites RNA-seq datasets were obtained from the GitHub repository and are also available at GEO: GSE64655. The *in vitro* mixture of immune cells was prepared by our laboratory and is available on our GitHub page. All supporting data and materials are available in the *GigaScience* GigaDB database [41].

## Additional Files

Supplementary Materials and Figures. Further details regarding synthetic mixture generation, deconvolution tool comparisons, and applications to the skin, mouse, and GTEx datasets.

Supplementary Table S1. Pairs of reference matrices used to generate synthetic mixtures.

Supplementary Figure S1. Error and correlation values when benchmarking is performed on 3 datasets (ascites, Hoek, and in vitro cell mixtures) using each of 5 tools (CIBERSORT, DeconRNASeq, dtangle, GEDIT, and xCell) and each of 4 possible reference matrices (BLUEPRINT, HPCA, ImmunoStates, LM22)

Supplementary Figure S2. Distribution of error values between predicted and actual fractions when deconvolution is applied to a set of 100 simulated pancreatic samples. Simulated samples are created using data single cell data from human pancreas, and separate single cell data is used to create a bulk reference matrix.

Supplementary Figure S3. CPU time for deconvolution tasks to complete when applied to inputs of varying size. Inputs are created by selecting varying numbers of samples from the GTEx database. For each input size, samples were randomly selected six times and deconvolution performed a total of 24 times, once for each of 4 tools. The LM22 matrix is used as a reference profile.

Supplementary Figure S4. Predicted cell type fractions for 21 skin samples using each of 4 tools. Samples represent microarray expression data from 21 biopsies of skin diseases, and the Skin Signatures matrix is used as a reference.

Supplementary Figure S5. Predicted cell type composition of 30 samples from the Mouse Body Atlas[6]. Samples were profiled using an Affymetrix U133A/GNF1H microarray. Single cell data from the Tabula Muris was averaged for each cell type to create a bulk reference.

Supplementary Figure S6. Cell types present in the 3 reference matrices used to predict cell type fractions of GTEX samples

Supplementary Figure S7. Clusters formed when the BluePrint, ENCODE, and Human Primary Cell Atlas (HPCA) matrices are combined, quantile normalized, then clustered. HPCA cell types do not cluster with similar cell types in BluePrint or ENCODE, and instead form an external cluster of only HPCA profiles. This is likely due to batch or platform specific effects. We explored using this combined reference to produce predictions for the GTEx database (see Supplementary Figure S8), but instead took a different approach, as described in the main paper.

Supplementary Figure S8. Results of GEDIT when applied to the GTEx database when using a combined reference from the Human Primary Cell Atlas, BluePrint, and ENCODE. Six reference profiles from the HPCA were added to the BlueCode reference matrix, then all profiles were quantile normalized. GEDIT was run on the 17,382 samples from the GTEx database. Predicted cell type composition is averaged for all samples of the same tissue (right side of graph).

## Abbreviations

CPM: Cell Population Mapping; DCQ: Digital Cell Quantifier; ENCODE: Encyclopedia of DNA Elements; FACS: fluorescence-activated cell sorting; GEDIT: Gene Expression Deconvolution Interactive Tool; GEO: Gene Expression Omnibus; GTEx: Genotype-Tissue Expression; MCP-Counter: microenvironment cell population counter; mRNA: messenger RNA; MuSiC: Multi-subject Single Cell deconvolution; NK: natural killer; SaVanT: Signature Visualization Tool; scRNA-seq: single-cell RNA sequencing; UCLA: University of California Los Angeles.

## Competing Interests

The authors declare that they have no competing interests.

## Funding

We acknowledge the Biomedical Big Data Grant (5T32LM012424–03) for supporting B.B.N. during the course of this research. We also acknowledge the Bruins-in-Genomics Summer Undergraduate Research Program for supporting H.W. and M.M.K. during the summer of 2017, when they contributed to this work. Whole-blood samples from healthy human donors were supplied through a sample-sharing agreement with the UCLA/CFAR Virology Core Lab (grant No. 5P30 AI028697). The Genotype-Tissue Expression (GTEx) Project was supported by the Common Fund of the Office of the Director of the National Institutes of Health, and by National Cancer Institute, National Human Genome Research Institute, National Heart, Lung, and Blood Institute, National Institute on Drug Abuse, National Institute of Mental Health, and National Institute of Neurological Disorders and Stroke.

## Authors' Contributions

BN developed the GEDIT tool and performed the evaluations of GEDIT and other deconvolution tools on test and real world datasets. DL provided guidance in initial development of GEDIT tool and assisted in construction of the GEDIT web portal. DJM titrated and sequenced the *in vitro* cell mixture used for evaluations. FM assembled the Tabula Muris reference dataset, using data from the original source [22]. HW and MMK assembled the 10x reference dataset, using data from the original source [20]. SM and MP provided oversight and assisted with manuscript editing and preparation.

## Supplementary Material

giab002_GIGA-D-20-00251_Original_Submission

giab002_GIGA-D-20-00251_Revision_1

giab002_Response_to_Reviewer_Comments_Original_Submission

giab002_Reviewer_1_Report_Original_Submission Jian Hu -- 9/10/2020 Reviewed

giab002_Reviewer_1_Report_Revision_1 Jian Hu -- 12/1/2020 Reviewed

giab002_Reviewer_2_Report_Original_Submission Paul Pavlidis, Ph.D. -- 9/16/2020 Reviewed

giab002_Supplemental_File
