# Supplementary material for: The Gene Expression Deconvolution Interactive Tool (GEDIT): accurate cell type quantification from gene expression data
Source: Gigascience. 2021 Feb 16;10(2):giab002. doi: 10.1093/gigascience/giab002 (PMC7931818; doi:10.1093/gigascience/giab002)
Supplement: giab002_Response_to_Reviewer_Comments_Original_Submission [file giab002_response_to_reviewer_comments_original_submission.pdf]

Author's Response To Reviewer Comments

Close

This following text is also included in the uploaded file "Response Letter":

Dear Giga Science Reviewer

Reviewer reports:

Reviewer #1: The authors present an algorithm called GEDIT using information from a reference dataset to estimate cell type abundances in a target dataset. This manuscript is not qualified to be published in the Giga Science because of the following reasons:
1. GEDIT does not show enough novelty.

As the authors stated in the response document, GEDIT has two key innovations: signature gene selection by information entropy and the row scaling step. As shown in Figure 2a, the signature gene selection only has a limited improvement than the others in terms of error. The authors also did not have enough evidence to support how and why the row scaling step is helpful. On top of these 2 data preprocessing steps, I did not find any innovations on the model of a non-negative linear regression.
We refer the reviewer to figure 4D, in which we demonstrate that row scaling dramatically improves accuracy of predictions. Here, we vary the row scaling parameter in order to evaluate the effect of row scaling on accuracy. When this parameter is set to 0 (the default value for GEDIT) both average and maximum error are greatly reduced compared to a setting of 1.0 (i.e. row scaling is disabled).

2. GEDIT does not add much values in the field.
Although GEDIT is shown to have appealing results in comparison to existing methods on benchmarking experiments, it doesn't significantly outperform other methods in real data analysis in regards to Pearson correlation and average error. As demonstrated in Supplementary Figures 4 and 5, a main determinant of results quality for deconvolution is the reference used, not necessarily the algorithm.

We refer to Figure 4, which demonstrates that GEDIT does indeed outperform other tools in terms of Pearson correlation. When any of the four references are used, GEDIT produces higher correlations between predicted and actual fractions than any other tool (the leftmost “all” column). In addition, the highest observed correlations for each of the 3 mixtures sets (ascites, cellmix, blood) are achieved by GEDIT (i.e. when the Human Primary Cell Atlas, LM22, and BLUEPRINT are used as reference sources, respectively). Lastly, unlike the three other reference-based tools, GEDIT produces positive correlations for all cell types regardless of choice of reference. GEDIT similarly outperforms other tools when evaluated by error (Supplementary Figure 2).
Moreover, we include in the supplementary materials an additional comparison of bulk deconvolution tools (Supplementary Figure 6). Here, we compare the error of CIBERSORT, DeconRNASeq, dtangle, and GEDIT when applied to simulated pancreatic islet mixtures. Again, GEDIT outperforms the other deconvolution tools.
In addition, we are pursuing a separate project performing extensive benchmarking of the current field of deconvolution tools. We believe more comprehensive comparisons of these tools’ performances is appropriate, but that such an undertaking represents a separate project that should not be bundled with the publication of a new tool. For the interest of the editors and reviewers, we attach an early version of this benchmarking manuscript.

3. I still think comparing GEDIT to other methods using single-cell RNA-seq as a reference is necessary. Utilizing single-cell RNA-seq for deconvolution becomes cutting-edge research and many tools have been designed for this purpose such as MuSic. These methods are proved to have superior performance using microarray as references and are commonly used. It is critical to demonstrate whether GEDIT has better performance than these methods.

At the reviewer’s request, we have performed a comparison between GEDIT and two well known single cell deconvolution tools (SCDC and MuSiC). Specifically, we utilize the testing framework developed by

the SCDC authors to prepare synthetic mixtures using single cell data from pancreatic islets. We include an additional section describing these results in the main manuscript (text below), and also refer the reviewer to Figure 5 and Supplementary Figure 5.

Comparison to Single Cell Methods
We also compare GEDIT to two contemporary deconvolution tools that utilize single cell data as their reference, namely SCDC and MuSiC [10,11]. We reproduce the steps provided by the SCDC authors to generate two sets of 100 simulated pancreatic mixtures. These data are created in silico using single cell data from two recent studies, and contain randomized mixtures of alpha, beta, delta, and gamma cells from pancreatic islets [33,34]. Data from a third study was used as a reference for all 3 tools, and similarly contains alpha, beta, gamma, and delta cells [35]. In the case of SCDC and MuSiC, these data are used in their original single cell form. For GEDIT, pseudo-bulk expression profiles for each of the four cell types were created by averaging the expression values of each member cell (e.g. expression of all alpha cells were averaged to create an alpha cell reference profile).
The results of GEDIT compare favorably to the two single cell tools (Figure 5). GEDIT produces the lowest error on the two sets of simulated mixtures by a significant margin. Based on the metric of correlation between predicted and actual fractions, GEDIT produces results comparable to SCDC, and either comparable or superior to MuSiC, depending on the set of mixtures (Table 4, Supplementary Figure 5). Thus, by using the methodology of averaging cell clusters in the reference dataset, GEDIT can be applied to datasets suitable for SCDC or MuSiC. We also apply three other bulk deconvolution tools to this same dataset, and show that GEDIT provides the best performance out of the four (Supplementary Figure 6).

Reviewer #2: For the most part the responses are sufficient and the authors have addressed the concerns, and the manuscript is improved. I especially appreciate that claims have been toned down and better contextualized.

A small issue remains about minor comment 6. My point was that the readers should be made clearly aware that doing three deconvolutions is not ideal, and strictly speaking invalid (e.g. cell contents totalling over 100%). I suggested two hypothetical ways to avoid this and am not at all surprised it's not easy to fix with data on hand. In the context of the demonstration in this particular study, what the authors did originally is acceptable. The problem I raised is if people start copying that practice in their own studies, and in the authors' own interest they presumably wouldn't want to be seen as endorsing it. The statement in the supplement that "creating a comprehensive reference from single cell data will likely produce superior results" should be more prominent, and it's not just about superior results, it's also about validity of having a single reference vs. multiple independent deconvolutions.

To be concrete, I'd suggest that in the main body a parenthetical could be added to the effect that "it would be more appropriate to have a single reference containing all cell types and performing a single deconvolution; see supplement for discussion". As it stands I don't think the addition to the supplement is referenced in the main paper.

We appreciate this feedback. We have adjusted the language in this section as follows:

To assess the use of GEDIT across very large datasets, we applied the tool to 17,382 GTEx RNA-seq samples collected from various tissues. However, no single reference contained all cell types expected to be present and combining references from separate experiments and platforms is problematic (Supplementary Figures 9-11). Therefore, we took an alternate approach by performing deconvolution three times using three separate references (BlueCode, Human Primary Cell Atlas, Skin Signatures). We then combine these outputs by taking their median value; after normalization, we treat this median value as a final cell type estimate (see Supplementary Materials for more details). While this approach did enable predictions spanning a larger number of cell types than are present in any one reference matrix, it must me noted that it is not a proper substitute for a single unified reference (Figure 8).

Close
