# Supplementary material for: The Gene Expression Deconvolution Interactive Tool (GEDIT): accurate cell type quantification from gene expression data
Source: Gigascience. 2021 Feb 16;10(2):giab002. doi: 10.1093/gigascience/giab002 (PMC7931818; doi:10.1093/gigascience/giab002)
Supplement: giab002_Reviewer_1_Report_Original_Submission — Jian Hu -- 9/10/2020 Reviewed [file giab002_reviewer_1_report_original_submission.pdf]

Reviewer Report

Title: The Gene Expression Deconvolution Interactive Tool (GEDIT): Accurate Cell Type Quantification from Gene Expression Data

Version: Original Submission **Date:** 9/10/2020

Reviewer name: Jian Hu

Reviewer Comments to Author:

â€¨The authors present an algorithm called GEDIT using information from a reference dataset to estimate cell type abundances in a target dataset. This manuscript is not qualified to be published in the Giga Science because of the following reasons:
1. GEDIT does not show enough novelty.
As the authors stated in the response document, GEDIT has two key innovations: signature gene selection by information entropy and the row scaling step. As shown in Figure 2a, the signature gene selection only has a limited improvement than the others in terms of error. The authors also did not have enough evidence to support how and why the row scaling step is helpful. On top of these 2 data preprocessing steps, I did not find any innovations on the model of a non-negative linear regression.
2. GEDIT does not add much values in the field.
Although GEDIT is shown to have appealing results in comparison to existing methods on benchmarking experiments, it doesn't significantly outperform other methods in real data analysis in regards to Pearson correlation and average error. As demonstrated in Supplementary Figures 4 and 5, a main determinant of results quality for deconvolution is the reference used, not necessarily the algorithm.
3. I still think comparing GEDIT to other methods using single-cell RNA-seq as a reference is necessary. Utilizing single-cell RNA-seq for deconvolution becomes cutting-edge research and many tools have been designed for this purpose such as MuSic. These methods are proved to have superior performance using microarray as references and are commonly used. It is critical to demonstrate whether GEDIT has better performance than these methods.

Methods

Are the methods appropriate to the aims of the study, are they well described, and are necessary controls included? Choose an item.

Conclusions

Are the conclusions adequately supported by the data shown? Choose an item.

Reporting Standards

Does the manuscript adhere to the journal's guidelines on [minimum standards of reporting?]() Choose an item.

Choose an item.

Statistics

Are you able to assess all statistics in the manuscript, including the appropriateness of statistical tests used? Choose an item.

Quality of Written English

Please indicate the quality of language in the manuscript: Choose an item.

Declaration of Competing Interests

Please complete a declaration of competing interests, considering the following questions:

- Have you in the past five years received reimbursements, fees, funding, or salary from an organisation that may in any way gain or lose financially from the publication of this manuscript, either now or in the future?
- Do you hold any stocks or shares in an organisation that may in any way gain or lose financially from the publication of this manuscript, either now or in the future?
- Do you hold or are you currently applying for any patents relating to the content of the manuscript?
- Have you received reimbursements, fees, funding, or salary from an organization that holds or has applied for patents relating to the content of the manuscript?
- Do you have any other financial competing interests?
- Do you have any non-financial competing interests in relation to this paper?

If you can answer no to all of the above, write 'I declare that I have no competing interests' below. If your reply is yes to any, please give details below.

I declare that I have no competing interests

I agree to the open peer review policy of the journal. I understand that my name will be included on my report to the authors and, if the manuscript is accepted for publication, my named report including any attachments I upload will be posted on the website along with the authors' responses. I agree for my report to be made available under an Open Access Creative Commons CC-BY license (http://creativecommons.org/licenses/by/4.0/). I understand that any comments which I do not wish to be included in my named report can be included as confidential comments to the editors, which will not be published.

Choose an item.

To further support our reviewers, we have joined with Publons, where you can gain additional credit to further highlight your hard work (see: https://publons.com/journal/530/gigascience). On publication of this paper, your review will be automatically added to Publons, you can then choose whether or not to claim your Publons credit. I understand this statement.

Yes Choose an item.
