# Supplementary material for: The Gene Expression Deconvolution Interactive Tool (GEDIT): accurate cell type quantification from gene expression data
Source: Gigascience. 2021 Feb 16;10(2):giab002. doi: 10.1093/gigascience/giab002 (PMC7931818; doi:10.1093/gigascience/giab002)
Supplement: giab002_Reviewer_2_Report_Original_Submission — Paul Pavlidis, Ph.D. -- 9/16/2020 Reviewed [file giab002_reviewer_2_report_original_submission.pdf]

Reviewer Report

Title: The Gene Expression Deconvolution Interactive Tool (GEDIT): Accurate Cell Type Quantification from Gene Expression Data

Version: Original Submission **Date:** 9/16/2020

Reviewer name: Paul Pavlidis, Ph.D.

Reviewer Comments to Author:

For the most part the responses are sufficient and the authors have addressed the concerns, and the manuscript is improved. I especially appreciate that claims have been toned down and better contextualized.
A small issue remains about minor comment 6. My point was that the readers should be made clearly aware that doing three deconvolutions is not ideal, and strictly speaking invalid (e.g. cell contents totalling over 100%). I suggested two hypothetical ways to avoid this and am not at all surprised it's not easy to fix with data on hand. In the context of the demonstration in this particular study, what the authors did originally is acceptable. The problem I raised is if people start copying that practice in their own studies, and in the authors' own interest they presumably wouldn't want to be seen as endorsing it. The statement in the supplement that "creating a comprehensive reference from single cell data will likely produce superior results" should be more prominent, and it's not just about superior results, it's also about validity of having a single reference vs. multiple independent deconvolutions.
To be concrete, I'd suggest that in the main body a parenthetical could be added to the effect that "it would be more appropriate to have a single reference containing all cell types and performing a single deconvolution; see supplement for discussion". As it stands I don't think the addition to the supplement is referenced in the main paper.

Methods

Are the methods appropriate to the aims of the study, are they well described, and are necessary controls included? Choose an item.

Conclusions

Are the conclusions adequately supported by the data shown? Choose an item.

Reporting Standards

Does the manuscript adhere to the journal's guidelines on [minimum standards of reporting?](#) Choose an item.

Choose an item.

Statistics

Are you able to assess all statistics in the manuscript, including the appropriateness of statistical tests used? Choose an item.

Quality of Written English

Please indicate the quality of language in the manuscript: Choose an item.

Declaration of Competing Interests

Please complete a declaration of competing interests, considering the following questions:

- Have you in the past five years received reimbursements, fees, funding, or salary from an organisation that may in any way gain or lose financially from the publication of this manuscript, either now or in the future?
- Do you hold any stocks or shares in an organisation that may in any way gain or lose financially from the publication of this manuscript, either now or in the future?
- Do you hold or are you currently applying for any patents relating to the content of the manuscript?
- Have you received reimbursements, fees, funding, or salary from an organization that holds or has applied for patents relating to the content of the manuscript?
- Do you have any other financial competing interests?
- Do you have any non-financial competing interests in relation to this paper?

If you can answer no to all of the above, write 'I declare that I have no competing interests' below. If your reply is yes to any, please give details below.

I declare that I have no competing interests

I agree to the open peer review policy of the journal. I understand that my name will be included on my report to the authors and, if the manuscript is accepted for publication, my named report including any attachments I upload will be posted on the website along with the authors' responses. I agree for my report to be made available under an Open Access Creative Commons CC-BY license (http://creativecommons.org/licenses/by/4.0/). I understand that any comments which I do not wish to be included in my named report can be included as confidential comments to the editors, which will not be published.

Choose an item.

To further support our reviewers, we have joined with Publons, where you can gain additional credit to further highlight your hard work (see: https://publons.com/journal/530/gigascience). On publication of this paper, your review will be automatically added to Publons, you can then choose whether or not to claim your Publons credit. I understand this statement.

Yes Choose an item.
